# Career Trajectory of Physicians Following a Fellowship Program: A Descriptive Study

**DOI:** 10.5041/RMMJ.10432

**Published:** 2021-04-29

**Authors:** Gidon Berger, Danny Epstein, Galit Kobi, Eyal Braun, Zaher S. Azzam, Michael Halberthal

**Affiliations:** 1 Department of Internal Medicine “B”, Rambam Health Care Campus, Haifa, Israel; 2 Hospital Management, Rambam Health Care Campus, Haifa, Israel; 3 Department of Internal Medicine “H”, Rambam Health Care Campus, Haifa, Israel; 4 Rappaport Faculty of Medicine, Technion–Israel Institute of Technology, Haifa, Israel

**Keywords:** Fellowship, graduate medical education, health professions education, medical career, medical education

## Abstract

**Introduction:**

A clinical and/or research fellowship abroad has become a prevalent choice among Israeli physicians. However, the influence of fellowship programs on the career path is unclear. We evaluated the role of physicians returning from fellowship in the organizational hierarchy and their professional and academic status.

**Methods:**

This was a retrospective, descriptive, cross-sectional study of physicians who completed a survey after accomplishing a fellowship. The survey included questions about the physicians’ attitudes toward the program, programs’ details, and the physicians’ current academic, professional, and administrative status. Information about scientific publications was also collected.

**Results:**

Of the 106 physicians receiving the questionnaire, 101 responded. The majority completed a two-year fellowship in North America. Forty percent participated in an integrated program (research and clinical), and 40% participated in clinical programs. Subjectively, the physicians attributed a significant value to the fellowship and positively recommend it. Most of the physicians held managerial positions, academic appointments, and had generated significant research.

**Discussion:**

The subjective perspective of all physicians participating in the study was that attending a fellowship program had a positive impact on their careers. Objectively, the accomplishment of a fellowship program empowered the studied physicians to become scholars, senior executives, and opinion leaders in their professional field.

## INTRODUCTION

Israel’s healthcare system is considered among the most advanced in the world. In many crucial parameters, Israel is ranked very high among the Organization for Economic Co-operation and Development (OECD) countries. For example, life expectancy is among the highest in the world, while infant mortality rates are among the lowest.^1^

As a relatively small nation, there are some limitations to the extent of medical education which Israeli hospitals and universities can provide. Therefore, Israeli physicians who finish residencies have difficulties moving to the “next phase” in their career if they wish to practice cutting-edge research. There are also limitations for physicians wanting to specialize in highly specific fields that mandate “large volume” centers to gain enough experience in the requested field.

From the hospital management perspective, fellowship programs are considered an important milestone, since the physicians return with clinical knowledge and experience, sometimes in new clinical fields. In addition, they acquire knowledge regarding advanced research methodologies and tools.

For these reasons, many Israeli residents travel abroad for fellowships, mainly in leading North American academic medical centers.

This study was aimed at describing the medical and academic career path of physicians who participated in international fellowship programs and examined their attitudes toward them.

## METHODS

This was a cross-sectional, retrospective, observational study, using an electronically mailed self-administered survey, targeting senior physicians who had completed a fellowship program in our hospital. Of the 323 senior physicians employed by our hospital, 106 had attended a fellowship program.

A web-based questionnaire was designed by the authors to investigate the evolution of physicians’ career paths after returning from a fellowship program, together with their subjective attitude toward the program (see Supplementary Material). Physicians’ subjective attitudes toward their fellowship program were measured using a rating scale of 1 to 7, with 7 being the most positive.

The survey included questions regarding physicians’ attitudes toward the attended fellowship program, as well as about fellowship program characteristics and funding, and the physicians’ career trajectory after the program, including their management role and academic and professional development. In addition to the survey, we collected information about publications by the physicians included in the study population. Data regarding the cohort’s publications were retrieved mainly from the US National Library of Medicine National Institutes of Health (PubMed), including the iCite database. The total number of citations for each physician was noted using the academic social network, ResearchGate.

During October 2019, a link to the digital questionnaire and a cover letter was e-mailed to 106 fellowship-graduated physicians. Non-responders were sent a reminder after one month. Participation was voluntary, with permission to participate in the study implied by the completion of the survey.

Survey results were summarized using descriptive statistics. Data analysis was conducted with Statistical Package for the Social Sciences, version 23.0 (SPSS, Chicago, IL, USA).

Under Israeli law, institutional review board approval was not required for this study.

## RESULTS

Of the 106 invitations sent to the study group, 101 questionnaires were returned, all of which were included in the study (response rate 95%). The mean age of participants was 52.72 (±9.04) years; 75.76% were males. The main fellowship program and the participants’ career characteristics are summarized in Table 1.

**Table 1:** Main Characteristics of the Fellowship Programs and the Career Characteristics of the 101 Study Participants.

Fellowship Parameter		*n* (%)
Country*	USA	66 (65.35%)
	Canada	21 (20.79%)
	European Union	13 (12.87%)
	Australia and New Zealand	2 (1.98%)
	Israel	2 (1.98%)

Specialty	Surgical specialties†	36 (35.64%)
	Internal medicine and subspecialties	40 (39.6%)
	Pediatrics	15 (14.85%)
	Anesthesia	4 (3.96%)
	Radiology	5 (4.95%)
	Psychiatry	1 (0.99%)

Duration‡	12 months or less	8 (7.92%)
	13 to 23 months	23 (22.77%)
	24 months or more	70 (69.31%)

Type	Research	20 (19.8%)
	Clinical	42 (41.58%)
	Combined	39 (38.61%)

**Career Parameter**		***n* (%)**

Current Position	Department Director	24 (23.76%)
	Deputy Director of Hospital	10 (9.9%)
	Unit Director	32 (31.68%)
	Senior Physician	34 (33.66%)

Academic Rank	Clinical Instructor	2 (1.98%)
	Lecturer	11 (10.89%)
	Senior Lecturer	16 (15.84%)
	Professor	27 (26.73%)

Funding	Our Institution	9 (8.91%)
	Host Institution	52 (51.49%)
	Self-funding	28 (27.72%)
	Other	12 (11.88%)

Publications (Median [IQR])	Number of Publications in PubMed	35 (15–76)
	Total Citations ResearchGate§	652 (223–1885)

* Three physicians participated in more than one fellowship program in different countries.

† Cardiothoracic surgery, general surgery, gynecology and obstetrics, gynecologic oncology, neurological surgery, ophthalmic surgery, oral and maxillofacial surgery, orthopedic surgery, plastic surgery, urology, and vascular surgery.

‡ Median length of fellowship: 2 years (IQR 1–3).

§ This information was available for 62 participants.

Of 99 responders, 80 (80.81%) strongly believed (grades 6 and 7) that a fellowship is an important part of their medical career; 76 of 100 (76%) responders strongly agreed that their professional development was influenced by the program. Only 27 of 95 (28.42%) responders felt that the hospital management encouraged physicians to take part in fellowship programs.

Of those surveyed, 100 of 101 (99%) indicated that they would recommend participation in a fellowship program to their colleagues; 89 (88.12%) believed that the fellowship had improved their clinical skills; 49 (48.51%) believed that it had improved their research skills.

## DISCUSSION

By the end of 2018, there were 15,989 specialist physicians under the age of 67 in Israel, with 1.78 per 1,000 population, similar to the OECD average. Specialist physicians make up about half of all physicians in the country, and 22% of them were aged below 45. One-fifth (22%) of the specialist physicians had two specialties.^2^

Many of the specialist physicians have traveled abroad to participate in a fellowship, mostly soon after completing their residencies. Fellowships are often self-financed, at least partially. Internal medicine specialists, in most cases, leave for a fellowship only after completing a residency in a sub-specialty.

For physicians planning a hospital career path, a fellowship program is a near-mandatory milestone. While some physicians attend one, to fulfill an unspecified expectation, others do so because a fellowship is a well-defined requirement from the hospital management. However, there is no coordination between the expectations of the physician and the hospital, nor are there defined goals for the physician who does attend a fellowship program, including no regular monitoring of his or her achievements during and after the program and no information on the physician’s personal professional development after completing it.

This study attempted to shed light on the personal professional development of physicians who participated in a fellowship program and describes the leadership, professional, and academic positions that Israeli physicians hold after returning from such programs. We also examined the physicians’ subjective attitude toward fellowship programs in general.

We believe these findings are relevant to other countries, mainly small ones with an advanced health system and high-quality manpower on the one hand, but limitations in diversity and wealth in the fields of research, medicine, and technology, on the other.

To date, two-thirds of the physicians who had participated in an overseas fellowship program (our study population) hold a management position in the hospital. In order to provide the full picture, we also examined the opposite and found that of the department heads and unit/service heads, 75% (30/40) and 64% (56/88), respectively, are graduates of a fellowship program. Based on these findings alone we cannot causally link a fellowship program to career success. Nevertheless, it is well known that many hospital administrations in Israel see participation in an overseas fellowship as an important step toward promotion within the organization.

Hospital administrations are well aware of the unique status and contribution of a physician-scientist and the potential of his/her impact on the organization.^3^ Another added value of the program in the eyes of hospital managers is the physician’s professional development during the fellowship in terms of clinical knowledge and skills that may support his/her status as a world-wide opinion leader, thereby leading to the addition of a new professional niche in the organization.

We found a high rate (54%) of academic appointments among the study population. The authors, and the study participants (based on their answers in the survey), believe that the fellowship made a significant contribution to this outcome, either directly, by engaging them in research during the program, or indirectly through observing the research activity and its significant place in leading organizations.^4^ Accordingly, we found a significant number of publications per physician, reflecting respectable research production. The impact of a research fellowship on subsequent publications has been shown previously by The Royal College of Surgeons of England and others.^5^,^6^ On the other hand, in the study of Agarwal et al., the h-index did not differ between fellowship- and non-fellowship-trained practitioners.^7^

Obviously, it was no coincidence to find both of these findings in our study population, since academic status significantly contributes to the advancement of a physician in our institute.

Many of the physicians in our study went on to become hospital leaders and the top in-hospital professional authority in their field, and 88% thought that the fellowship program had contributed to their professional development.

In line with what is mentioned above, and consistent with previous reports about fellowship program graduates’ satisfaction, we found a very positive attitude toward the program and its beneficial effects on the physicians’ career.^8^ Ninety-nine percent answered positively to the question “Would you recommend that a young physician participate in a fellowship program?”

Another component of the program with a significant contribution to the physicians’ future is the “social” component. Networking and collaborating with colleagues, based on personal acquaintance during the program, play a significant role in the physician’s career, professionally and academically.^9^ However, this was not quantified and evaluated in our study. Nevertheless, we can point to close working relationships and joint research projects between many Israeli physicians in our study population, and their overseas colleagues.

## LIMITATIONS

This study was conducted in a single center that has a strong research orientation and places a particular emphasis on attending a fellowship program. In addition, the study did not include a control group, and therefore a causal relationship between the program and the career path could not be established. It is important to note that in any case it was not possible to prove a causal link, as it is impossible to neutralize the “selection bias” of the physicians who choose to leave for a fellowship. On the basis of our preliminary results, it is possible to advance to a larger study at the national level, which will include centers with different characteristics and control groups.

## CONCLUSION

In summary, physicians believe that fellowship programs make a significant contribution to their careers. Objectively, it seems that attending a fellowship program provides added value to physicians and assists in their career development.

## Supplementary Information



## Abbreviations

OECD: Organization for Economic Co-operation and Development.
