# Supplementary material for: Career Trajectory of Physicians Following a Fellowship Program: A Descriptive Study
Source: Rambam Maimonides Med J. 2021 Apr 29;12(2):e0011. doi: 10.5041/RMMJ.10432 (PMC8092956; doi:10.5041/RMMJ.10432)
Supplement: Supplementary file 1 [file rmmj-12-2-e0011-AM.docx]

This appendix has been provided by the authors for the benefit of readers

Supplement to Career Trajectory of Physicians Following a Fellowship Program: A Descriptive Study

Berger G, Epstein D, Kobi G, Braun E, Azzam ZS, Halberthal M. Career Trajectory of Physicians Following a Fellowship Program: A Descriptive Study. Rambam Maimonides Med J 2020;12 (2):e0011. doi:10.5041/RMMJ.10432

# Translation of Questions Asked of Study Participants

1. Do you believe that a fellowship program is a significant part of your medical career?
2. Do you agree that your professional and academic development was influenced by the program?
3. Did you feel that the hospital management encouraged you to take part in the fellowship program?
4. Would you recommend participation in a fellowship program to your colleagues/young physicians?
